# Progress of Conductivity and Conduction Velocity Measured in Human and Animal Hearts

**DOI:** 10.31083/j.rcm2510364

**Published:** 2024-10-11

**Authors:** Zhenyin Fu, Ruiqing Dong, Huanyong Zheng, Zefeng Wang, Boyang Cao, Jinghui Bai, Mingxia Ma, Zhanchun Song, Fuzhi Pan, Ling Xia, Yongquan Wu, Shijie Zhou, Dongdong Deng

**Affiliations:** ^1^College of Biomedical Engineering & Instrument Science, Zhejiang University, 310058 Hangzhou, Zhejiang, China; ^2^Department of Cardiology, Dushu Lake Hospital Affiliated to Soochow University, 215000 Suzhou, Jiangsu, China; ^3^School of Biomedical Engineering, Dalian University of Technology, 116024 Dalian, Liaoning, China; ^4^Department of Cardiology, Beijing Anzhen Hospital Affiliated to Capital Medical University, 100029 Beijing, China; ^5^Department of General Medicine, Liaoning Cancer Hospital of Dalian University of Technology, 116024 Liaoning, China; ^6^Department of Cardiology, Fushun Central Hospital, 113006 Liaoning, China; ^7^Research Center for Healthcare Data Science, Zhejiang Lab, 310058 Hangzhou, Zhejiang, China; ^8^Department of Chemical, Paper and Biomedical Engineering, Miami University, Oxford, OH 45056, USA

**Keywords:** isthmus, outer loop, conduction velocity, conductivity, anisotropic ratio

## Abstract

Cardiac conduction velocity (CV) is a critical electrophysiological characteristic of the myocardium, representing the speed at which electrical pulses propagate through cardiac tissue. It can be delineated into longitudinal, transverse, and normal components in the myocardium. The CV and its anisotropy ratio are crucial to both normal electrical conduction and myocardial contraction, as well as pathological conditions where it increases the risk of conduction block and reentry. This comprehensive review synthesizes longitudinal and transverse CV values from clinical and experimental studies of human infarct hearts, including findings from the isthmus and outer loop, alongside data derived from animal models. Additionally, we explore the anisotropic ratio of conductivities assessed through both animal and computational models. The review culminates with a synthesis of scientific evidence that guides the selection of CV and its corresponding conductivity in cardiac modeling, particularly emphasizing its application in patient-specific cardiac arrhythmia modeling.

## 1. Introduction

Cardiac conduction velocity (CV) is a critical electrophysiological characteristic of the myocardium, describing the rate at which electrical pulses propagate through cardiac tissue. This velocity is notably reduced in diseased myocardium compared to a healthy state [1, 2, 3]. Slowed conduction in the myocardium predisposes the formation of reentrant circuits by facilitating unidirectional blocks, which are critical for initiating reentry. Once initiated, these reentrant circuits can sustain arrhythmias, creating a substrate for the occurrence of a unidirectional block, continuously disrupting normal heart rhythms [4, 5]. Therefore, the precise quantification of CV in both healthy and pathological hearts is essential for investigating the mechanisms underpinning the initiation and maintenance of cardiac arrythmias [3, 6].

In human myocardium, CV is characterized by longitudinal (CV_l_), transverse (CV_t_) and normal (CV_n_) components [1]. These components reflect the directional dependency, or anisotropy, of electrical propagation relative to myocardial fiber orientation [7, 8]. The electrical pulse travels faster along the longitudinal direction than the transverse and normal direction in myocardial fibers. Changes in CV and its anisotropic ratios play an important role in electrical conduction and myocardial contraction in normal and disease states. Therefore, accurately characterizing these changes in CV and conductivity under different physiological and pathological conditions is crucial for developing computational models aimed at investigating cardiac electrical remodeling and arrhythmogenesis.

It has been demonstrated that conductivity values are critical for cardiac modeling, which is essential for simulating various bioelectric phenomena [9, 10, 11, 12, 13]. For instance, variations in myocardial conductivity can significantly influence the outcomes of computational models, as evidenced by research using heart models from different species. In a notable study, Sampson and Henriquez [9] utilized mouse and rabbit heart models with conductivity values ranging from 0.125 mS/cm to 4.0 mS/cm. Their results demonstrated that as the conductivity decreased, the dispersion of action potential duration increased [9]. Similarly, Bishop *et al*. [10] incorporated different intra- and extracellular conductivities in a rabbit heart model, observing distinct impacts on electrical signal propagation. Specifically, the intracellular conductivity was 1.70 mS/cm along the fiber and 0.19 mS/cm along the cross-fiber direction, while the extracellular conductivity was 6.20 mS/cm and 2.40 mS/cm, respectively [10]. Prakosa *et al*. [11] employed longitudinal and transverse conductivities of 2.55 mS/cm and 0.775 mS/cm, respectively, in their simulation of post-infarct ventricular tachycardia (VT). In separate studies by Carpio *et al*. [12, 13], the longitudinal and transversal conductivity values were set to 5.00 mS/cm and 1.00 mS/cm, respectively. These specific examples highlight the importance of selecting appropriate conductivity values in mathematical models, ensuring realistic and precise simulations of cardiac bioelectric phenomena.

The selection of conductivity values for cardiac modeling shows considerable variability, even for same species or under similar physiological and pathological conditions. This variability can significantly impede the reproducibility and validation of simulation results. Addressing this challenge necessitates the establishment of a standardized range of CV and conductivity values, applicable under both physiological and pathological conditions. Such standardization would facilitate a more accurate characterization of alterations in CV and conductivity, enhancing the capability of models to investigate the underlying mechanisms of cardiac electrical remodeling and arrhythmogenesis effectively.

This review aims to synthesize data on cardiac CV in both the isthmus and outer loop of human infarct hearts and across various animal models. The secondary objective is to collate findings on the anisotropic ratios of conductivity derived from experimental measurements and numerical simulations. Ultimately, this review seeks to provide scientific evidence to guide the selection of CV and the corresponding conductivity in cardiac modeling or other applications. By elucidating the relationship between CV and cardiac arrhythmias, this comprehensive analysis may ultimately foster the development of more effective diagnostic and therapeutic interventions for patients with cardiac disease, potentially enhancing patient outcomes.

## 2. CV in the Isthmus Region of Human Infarcted Hearts: Clinical Measurements

**Fig. 1.**
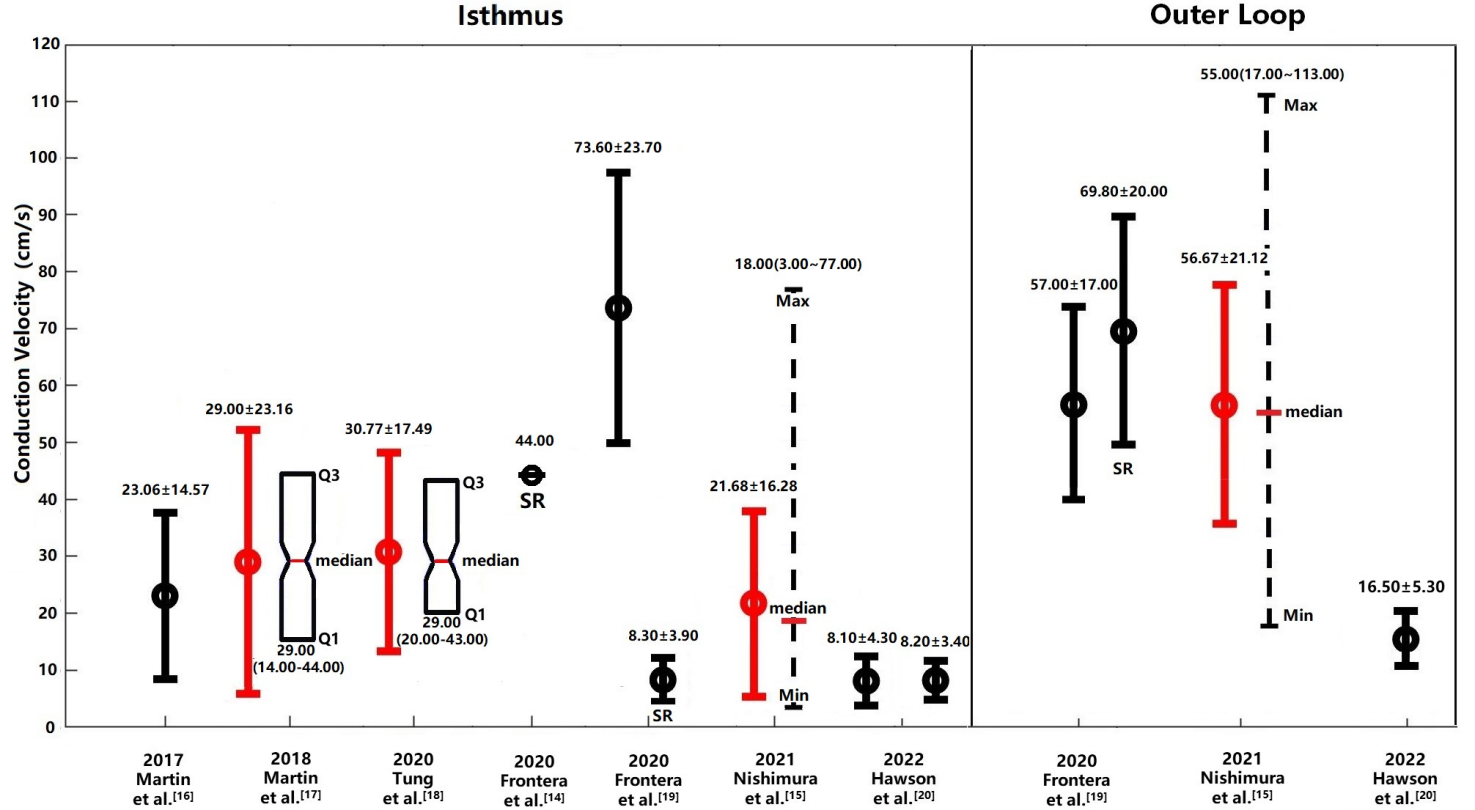
**Cardiac CVs in the isthmus region and outer loop of the human heart under clinical conditions**. CVs measured in the isthmus and outer loop regions of the human heart under clinical conditions are depicted. CV values obtained during SR are shown unless otherwise indicated, with measurements during VT presented where SR data are unavailable. Values originally reported in the literature are represented in black, while those transformed to mean ± SD for comparative analysis are shown in red. Abbreviations: SR, sinus rhythm; Min, minimum; Max, maximum; Q1, 1st quartile; Q3, 3rd quartile; CV, conduction velocity; VT, ventricular tachycardia.

In clinical practice, the CV of the entrance, exit, and isthmus regions within the infarcted areas of the human heart are commonly measured to diagnose and treat VTs. Notably, some studies [14, 15] have extended these measurements to the outer loop, which is a critical component of the reentrant VT circuit. This review aims to consolidate the CV measured from these diverse regions to construct a comprehensive profile of the VT circuit. While some research groups have presented their CV data as mean and standard deviation (SD), others have employed a five-number summary method that includes the sample median, the first and third quartiles, and the minimum and maximum values. For uniformity in comparison, this article has converted CV values from the five-number summary to mean ± SD, where feasible. Instances where conversion was impeded by incomplete data retain their original format as cited in the references. Table 1 (Ref. [14, 15, 16, 17, 18, 19, 20]) summarizes the longitudinal CVs measured in different regions, and Fig. 1 illustrates these CVs in the isthmus region and outer loop, represented by mean ± SD method and as originally reported.

**Table 1.** **Longitudinal CV of human hearts: clinical measurements (unit: cm/s)**.

Years	Study	Status	CV type	No.	Entrance	Exit	Isthmus	Central	Outer loop	SCinOL	Dead end	VSCIB
2019	Martin *et al*. [16]	VT	Mean ± Std	7	7.70 ± 1.63	11.63 ± 6.85	23.06 ± 14.57	-	-	-	-	-
2018	Martin *et al*. [17]	VT	Median (min–max)	31	8.00 (6.00–12.00)	11.00 (7.00–22.00)	29.00 (14.00–44.00)	-	-	-	21.00 (1.00–49.00)	6.00 (4.00–10.00)
2020	Tung *et al*. [18]	VT	Median (min–max)	97	33.00	37.00	29.00 (20.00–43.00)	19.00 (13.00–29.00)	-	-	-	-
2020	Frontera *et al*. [14]	SR	Mean	16	10.00	17.00	44.00	-	-	-	-	-
2020	Frontera *et al*. [19]	VT	Mean ± Std	6	8.80 ± 3.90	7.00 ± 3.50	73.60 ± 23.70	-	57.00 ± 17.00	20.30 ± 7.90	-	-
2020	Frontera *et al*. [19]	SR	Mean ± Std	6	-	-	8.30 ± 3.90	-	69.80 ± 20.00	20.60 ± 7.90	-	-
2021	Nishimura *et al*. [15]	VT	Median (min–max)	49	16.00 (3.00–55.00)	14.00 (3.00–53.00)	18.00 (3.00–77.00)	26.00 (6.00–81.00)	55.00 (17.00–113.00)	-	-	-
2022	Hawson *et al*. [20]	VT	Mean ± Std	15	8.00 ± 3.60	16.20 ± 0.97	8.10 ± 4.30	-	16.50 ± 5.30	-	-	-
					10.40 ± 3.50	8.20 ± 3.50	8.50 ± 3.40	-	-	-	-	-

No., number of patients; SCinOL, slow conduction in outer loop; VSCIB, very slow conduction in isthmus barriers; min, minimum; max, maximum; VT, ventricular tachycardia; SR, sinus rhythm; CV, conduction velocity; Std, standard deviation.

In 2019, Martin *et al*. [16] documented CVs in patients with VTs, finding that the CV at the center of the isthmus region averaged 23.06 ± 14.57 cm/s in 7 patients, while the entrance and exit regions exhibited lower CVs of 7.70 ± 1.63 cm/s and 11.63 ± 6.85 cm/s, respectively. A subsequent study in 2018 by Martin *et al*. [17] measured the median CV at the entrance, exit and isthmus regions of the VT circuit in 31 patients with complex VT circuits, involving multiple entrances, exits, and dead ends. They reported a median CV of 8.00 cm/s at the entrance zone, 29.00 cm/s at the isthmus region, and 11.00 cm/s at the exit region [17]. For analytical consistency, the isthmus region values were also transformed into mean and SD of 29.00 cm/s ± 23.16 cm/s.

In 2020, Tung *et al*. [18] conducted a study on the 3-dimentional (3D) human VT circuit using simultaneous endocardial and epicardial mappings. They reported a median CV of 29.00 cm/s at the isthmus and 19.00 cm/s at the central isthmus, with transformed mean and SD at the isthmus region of 30.77 cm/s ± 17.49 cm/s respectively. Additionally, the median CV at the entrance and exit regions were 8.00 cm/s and 11.00 cm/s, respectively. In a related investigation, Frontera *et al*. [14] measured the mean CV during sinus rhythm to be 44.00 cm/s at the isthmus region in 16 patients, while the mean CV at the entrance and exit regions were 10.00 cm/s and 17.00 cm/s respectively. In another study, the same group [19] measured the CV in the outer loop during VT and sinus rhythm. The CV (mean ± SD) during VT at the entrance, exit and central isthmus were 8.80 ± 3.90 cm/s, 7.00 ± 3.50 cm/s and 73.60 ± 23.70 cm/s, respectively [19]. Meanwhile, during sinus rhythm, the CV at the isthmus region was 8.30 ± 3.90 cm/s [19].

In 2021, Nishimura *et al*. [15] assessed the determinants of VT cycle length (CL) in patients with both stable and unstable VT using high-resolution multielectrode mapping. They reported median CVs of 16.00 cm/s, 14.00 cm/s, 18.00 cm/s and 26.00 cm/s at entrance, exit, isthmus and mid-50% isthmus, respectively [15]. The mean and SD at isthmus region were 21.68 ± 16.28 cm/s, after transformation from five-number summary. The following year, Hawson *et al*. [20] measured CVs using both automated conduction velocity mapping (ACVM) and traditional substrate mapping. The ACVM method yielded CVs of 8.00 ± 3.60 cm/s, 16.20 ± 0.97 cm/s, 8.10 ± 4.30 cm/s and 16.50 ± 5.30 cm/s at entrance, exit, mid-isthmus and outer loop, respectively [20]. In contrast, traditional mapping produced CVs of 10.40 ± 3.50 cm/s, 8.20 ± 3.50 cm/s and 8.20 ± 3.40 cm/s at entrance, exit and mid-isthmus, respectively [20].

## 3. CV in the Outer Loop Region of Human Infarcted Hearts: Measurements from Clinical and Experimental Studies

Historically, research on the VT circuit have primarily focused on the isthmus, often neglecting the outer loop (OL) [15, 19]. The OL is defined as the shortest distance from the exit to the entrance along the reentrant wave-front [19]. However, findings by Frontera *et al*. [19] in 2020 challenged this perspective by demonstrating that the OL is not only a passive participant but also a critical substrate for VT. They observed that during VT, the CV in the OL and the slow OL conduction region were 57.00 ± 17.00 cm/s and 20.30 ± 7.90 cm/s, respectively [19]. Interestingly, during sinus rhythm, these values shifted to 69.80 ± 20.00 cm/s and 20.60 ± 7.90 cm/s, respectively, indicating dynamic changes in conduction based on the cardiac state [19]. Further emphasizing the OL’s importance, Nishimura *et al*. [15] in 2021 identified that the CV of the OL, rather than the isthmus, is the primary determinant of the rate of VT, with a transformed mean and SD of 56.67 ± 21.12 cm/s in the OL. These significant findings are visually summarized in Fig. 1 illustrating the CVs in the OL, represented by mean ± SD and/or original value from the published reference.

## 4. Pathophysiology of Slow Conduction at the Entrances and Exits of VT Circuit Isthmus

The pathophysiology of slow conduction at the entrance and exit of the VT circuit isthmus involves an intricate interplay of structural and electrical remodeling within myocardial tissue [21]. Key factors affecting electrical conduction include cellular excitability and connectivity: the cellular excitability is primarily determined by the functional status of cardiac sodium channels, while connectivity is influenced by connexin expression and structural alterations caused by fibrosis [22]. In the context of VT, scarred or fibrotic tissue, often resulting from myocardial infarction, creates an anatomical substrate that impedes normal electrical propagation, thus diminishing conduction velocities and promoting the development of re-entry circuits [22]. Specifically, the slowest CV at the entrance and exit of the VT circuit isthmus are attributable to changes in wave front curvature, increased axial resistivity, and thickness gradients [23, 24, 25]. Additionally, an important factor may be the discontinuous fiber orientation at the boundaries between infarcted and non-infarcted tissues, which are typically located at the entrances and exits of the VT circuit, further slowing CV at these critical junctures [26, 27].

## 5. CV Anisotropy in Human Cardiac Tissue: Comparing Longitudinal and Transverse Measurements

**Fig. 2.**
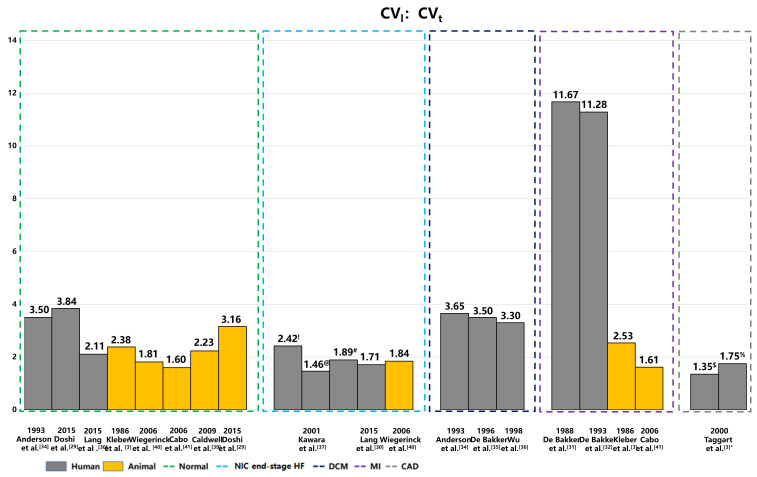
**Anisotropic ratios of CV in human and animal hearts under healthy and pathological conditions**. This figure illustrates the anisotropic ratios of longitudinal and transverse CVs across a spectrum of heart diseases, including coronary artery disease with MI, hypertrophic cardiomyopathy, and DCM. The data highlights distinct patterns in CV anisotropy corresponding to different types of myocardial fibrosis—patchy, diffuse, and stringy. These visualizations underscore the significant impact of fibrotic remodeling on electrical propagation within the heart. The yellow color represents the CV anisotropic ratio of animal hearts, and the gray color represents human hearts. NIC end-stage HF, nonischemic end-stage heart failure; DCM, dilated cardiomyopathy; MI, myocardial infarction; CAD, coronary artery disease. ^!^: Diffuse fibrosis; ^@^: Patchy fibrosis; ^#^: Stringly fibrosis; ^$^: CAD; ^%^: CAD with early ischemia; CV, conduction velocity; CV_t_, transverse CV; CV_l_, longitudinal CV.

While clinical studies commonly measure the longitudinal CV in the heart, it is important to note that the electrical conduction is not isotropic [28]. Therefore, it is crucial to measure the appropriate anisotropy of both longitudinal and transverse CVs in both healthy and diseased hearts [28]. The directionality of myocyte fiber largely determines the electrical conduction rate, with longitudinal conduction being faster than transverse conduction, resulting in an anisotropic activation pattern [29]. Therefore, it is crucial to measure the appropriate anisotropy of both longitudinal and transverse CVs in both healthy and diseased hearts. Table 2 (Ref. [29, 30, 31, 32, 33, 34, 35, 36, 37]) provides a summary of the longitudinal and transverse CVs of the human heart, as measured from experimental studies. Furthermore, Fig. 2 illustrates the anisotropic ratio of CV in human hearts under both healthy and diseased conditions.

**Table 2.** **Summary of longitudinal and transverse CV of human hearts under healthy and pathological conditions**.

Disease type	Year	Study	Tissue	CV_l_	CV_t_	CV_l_/CV_t_
Control	1993	Anderson *et al*. [34]	LV epi	80.00 ± 8.00	23.00 ± 3.00	3.50
	2015	Doshi *et al*. [29]	LV	56.10	14.60	3.84
	2015	Lang *et al*. [30]	LV	95.00 ± 5.00	45.00 ± 5.00	2.11
MI	1988	de Bakker *et al*. [31]	Papillary muscle	70.00	6.00	11.67
	1993	de Bakker *et al*. [32]	Papillary muscle	79.00	7.00	11.28
CAD	2000	Taggart *et al*. [33]	LV free wall	65.00	48.00	1.35
CAD with early ischemia				56.00	32.00	1.75
DCM	1993	Anderson *et al*. [34]	LV epi	84.00 ± 9.00	23.00 ± 5.00	3.65
	1996	de Bakker *et al*. [35]	Papillary muscle	70.00	20.00	3.50
	1998	Wu *et al*. [36]	Ventricle	66.00 ± 9.00	<20.00	3.30
NIC end-stage HF	2001	Kawara *et al*. [37]	Ventricle: Diffuse fibrosis	58.00 ± 15.00	24.00 ± 4.00	2.42
			Patchy fibrosis	57.00 ± 13.00	39.00 ± 13.00	1.46
			Stringy fibrosis	53.00 ± 19.00	28.00 ± 7.00	1.89
	2015	Lang *et al*. [30]	LV	60.00 ± 20.00	35.00 ± 15.00	1.71

MI, myocardial infarction; CAD, coronary artery disease; DCM, dilated cardiomyopathy; NIC end-stage HF, nonischemic end-stage heart failure; CV, conduction velocity; CV_t_, transverse CV; CV_l_, longitudinal CV; LV, left ventricle.

The longitudinal and transverse CVs in diseased hearts can vary significantly. In a 2015 study, Doshi *et al*. [29] used high-density optical and electrical mapping methods to measure CV in a healthy nonfailing donor heart, recording a longitudinal CV of 56.10 cm/s and a transverse CV of 14.60 cm/s, resulting in an anisotropic ratio of 3.8. Another study in the same year by Lang *et al*. [30] estimated the longitudinal and transverse CVs in 8 nonfailing donor hearts using the optical mapping, which showed an average longitudinal CV of 95.00 ± 5.00 cm/s and an average transverse CV of 45.00 ± 5.00 cm/s, with an anisotropic ratio of 2.11. These findings highlight the importance of measuring both longitudinal and transverse CVs in healthy and diseased hearts to fully understand the anisotropic activation patterns. Such measurements are crucial for identifying and treating cardiac arrhythmia.

Studies have shown significant variation in the longitudinal and transverse CVs of diseased hearts. For example, de Bakker *et al*. [31, 32] utilized intraoperative and optical mapping methods to measure the longitudinal and transverse CVs in the papillary muscle of human hearts affected by myocardial infarction (MI). They reported longitudinal CV values of 70.00 cm/s and 79.00 cm/s, alongside transverse CV values of 6.00 cm/s and 7.00 cm/s, and anisotropic ratios of 11.67 and 11.28, respectively [31, 32]. Taggart *et al*. [33] used plunge electrode recordings to measure the anisotropic CV in patients with coronary artery disease, reporting longitudinal and transverse CV values of 65 cm/s and 48 cm/s, respectively, under control conditions. When these values were assessed during early ischemia, the longitudinal CV decreased slightly, while transverse CV slowed substantially (56.00 cm/s vs. 32.00 cm/s) [33]. In patients with dilated cardiomyopathy (DCM), previous histopathologic studies revealed significant interstitial replacement and perivascular fibrosis in the human ventricles [38]. This excessive fibrous tissue between myofibrils and bundles can lead to inhomogeneous anisotropy, potentially enhancing electrolytic coupling. In 1993, Anderson *et al*. [34] used an electrode array to measure longitudinal and transverse CVs in 15 DCM patients, reporting values of 84.00 ± 9.00 cm/s and 23.00 ± 3.00 cm/s, respectively.

Later, de Bakker *et al*. [35] utilized high-resolution mapping to measure the electrical activity of 7 patients who underwent cardiac transplantation for DCM. They observed longitudinal and transverse CVs of 70.00 cm/s and 20.00 cm/s, respectively, with an anisotropic ratio of 3.5 [35]. Similarly, in 1998, Wu *et al*. [36] employed an electrode array to evaluate CV in five patients with DCM and severe congestive heart failure, reporting a longitudinal CV of 66.00 ± 9.00 cm/s and a transverse CV of less than 20.00 cm/s, resulting in an anisotropic conduction velocity ratio greater than 2.5.

In 2001, Kawara *et al*. [37] conducted high-resolution unipolar mapping in 11 human hearts afflicted with various diseases, including coronary artery disease with MI, hypertrophic cardiomyopathy, DCM. They noted that longitudinal CVs were consistent in the three types of fibrosis: 58.00 ± 15.00 cm/s for patchy, 57.00 ± 13.00 cm/s for diffuse, and 53.00 ± 19.00 cm/s for stringy fibrosis [37]. However, marked differences were observed in the anisotropy in transverse CVs, which were 24.00 ± 4.00 cm/s for diffuse, 39.00 ± 13.00 cm/s for patchy, and 28.00 ± 7.00 cm/s for stringy fibrosis [37]. Additionally, in their study on heart failure patients, Lang *et al*. [30] reported the longitudinal and transverse CVs were 60.00 ± 10.00 cm/s and 35.00 ± 15.00 cm/s, respectively, underscoring the impact of cardiac health on electrical conduction patterns.

## 6. CV Anisotropy in Animal Cardiac Models: Comparing Longitudinal and Transverse Measurements

In a pivotal study, Kléber *et al*. [3] utilized a pig model of acute ischemia to assess the longitudinal and transverse CVs. During the acute ischemia condition, longitudinal CVs decreased to 38.00 ± 3.00 cm/s from 50.08 ± 2.13 cm/s while the transverse CVs decreased to 15.00 ± 1.00 cm/s from 21.08 ± 0.97 cm/s while the anisotropic ratio increased to 2.53 from 2.38 [3]. Complementing these findings, Caldwell *et al*. [39] in 2009 provided additional data from 5 pig hearts, showing CVs of 67.00 ± 1.90 cm/s, 30.00 ± 1.00 cm/s and 17.00 ± 0.40 cm/s respectively under controlled experimental conditions. These studies collectively highlight the dynamic changes in cardiac electrophysiology under varying physiological stresses and their implications for cardiac health. Table 3 (Ref. [3, 29, 39, 40, 41]) presents a summary of longitudinal, transverse and normal CVs of animal hearts measured from experimental studies, while Fig. 2 shows the anisotropic CV ratio of animal hearts in healthy and diseased conditions.

**Table 3.** **Longitudinal, transverse and normal CV of animal hearts across different experimental conditions (unit: cm/s)**.

Disease type	Year	Study	Tissue	Normal area (cm/s)				Diseased area (cm/s)			
				CV_l_	CV_t_	CV_n_	CV_l_/CV_t_	CV_l_	CV_t_	CV_n_	CV_l_/CV_t_
Acute ischemia (Pig)	1986	Kléber *et al*. [3]	Isolated heart	50.08 ± 2.13	21.08 ± 0.97	-	2.38	38.00 ± 3.00	15.00 ± 1.00	-	2.53
NIC end-stage HF (Rabbit)	2006	Wiegerinck *et al*. [40]	LV free wall	67.00 ± 4.00	37.00 ± 2.00	-	1.81	79.00 ± 2.00	43.00 ± 2.00	-	1.84
MI (Canine)	2006	Cabo *et al*. [41]	LV	45.00 ± 7.00	28.00 ± 5.00	-	1.60 ± 0.30	29.00 ± 5.00	18.00 ± 5.00	-	1.61 ± 0.30
Normal (Pig)	2009	Caldwell *et al*. [39]	LV free wall	67.00 ± 2.00	30.00 ± 1.00	17.00 ± 0.40	2.23	-	-	-	-
Normal (Mouse)	2015	Doshi *et al*. [29]	LV	53.10	16.80	-	3.16	-	-	-	-

NIC end-stage HF, nonischemic end-stage heart failure; MI, myocardial infarction; CV, conduction velocity; CV_t_, transverse CV; CV_l_, longitudinal CV; LV, left ventricle; CV_n_, normal CV.

In 2006, Wiegerinck *et al*. [40] measured the longitudinal and transvers CVs in a rabbit model of heart failure. Under the control condition, the longitudinal and transverse CVs were 67.00 ± 4.00 cm/s and 37.00 ± 2.00 cm/s, respectively. Remarkably, during heart failure, these CVs increased to 79.00 ± 2.00 cm/s and 43.00 ± 2.00 cm/s, respectively, with the anisotropic ratio slightly rising from 1.81 to 1.84 [40]. This unusual increase in CVs during heart failure is primarily attributed to cellular hypertrophy, characterized by an increase in cell size [40]. In the moderate HF model employed by Wiegerinck *et al*. [40], electrical remodeling factors, such as alterations in the sodium current and fibrosis, are not present. The absence of confounding factors during the cardiac remodeling processes renders highlights the applicability of the rabbit model, particularly for isolating the effects of hypertrophy on CVs. This insight is vital for developing targeted therapies for heart failure that directly address alterations in cardiac conduction properties. Additionally, in a separate study on transgenic mouse hearts, Doshi *et al*. [29] reported the longitudinal and transverse CVs to be 53.10 cm/s and 16.80 cm/s, respectively, with an anisotropic CV ratio of 3.16. This comparison further illustrates the variability of conduction properties across different animal models and cardiac conditions.

## 7. Experimental Conductivity Values

**Fig. 3.**
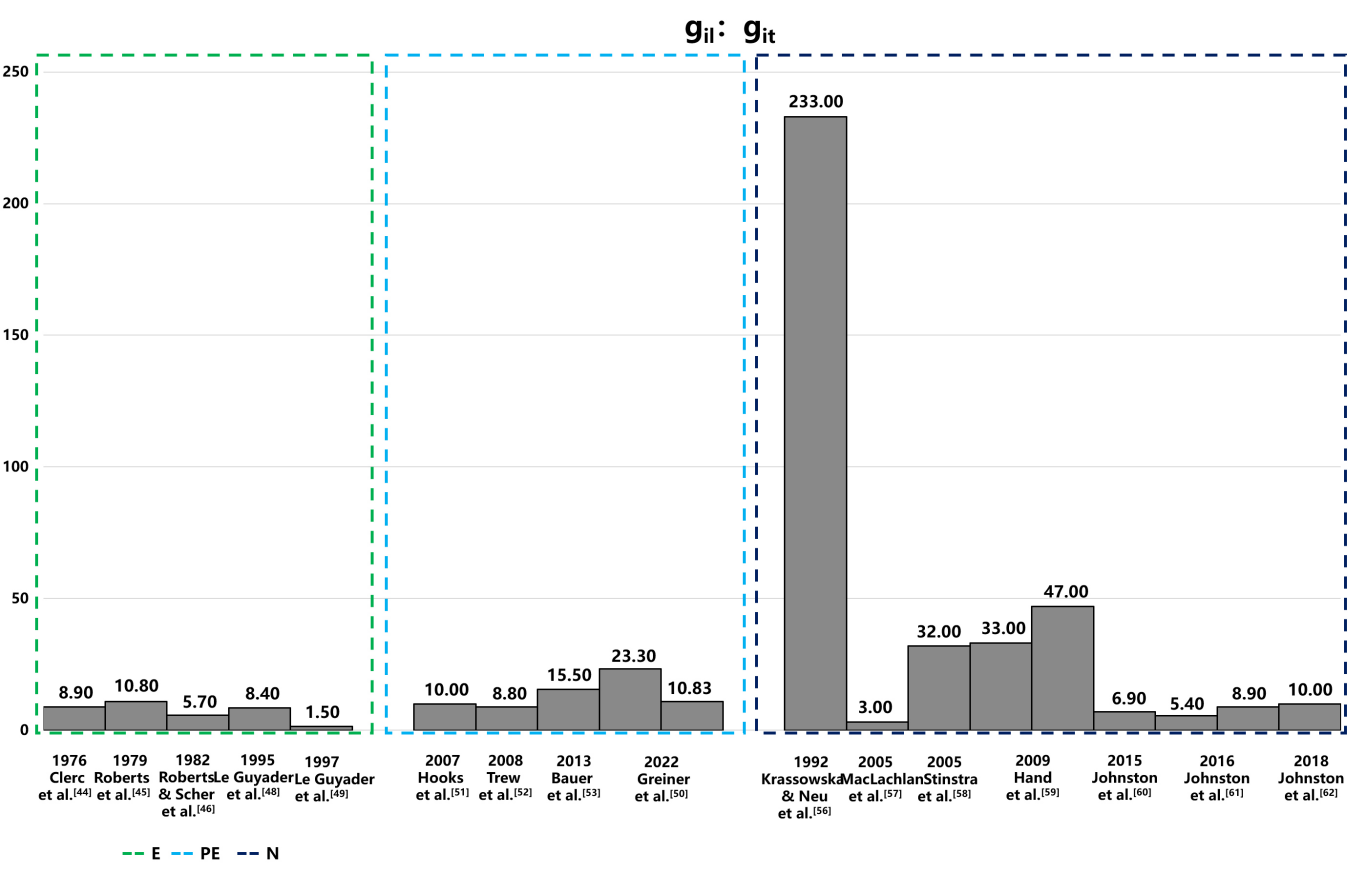
**Comparison of intracellular conductivity values derived from experimental measurements and numerical simulation**. This figure presents intracellular conductivity values, showcasing variations between those directly measured in experiments and those estimated via numerical simulations. Notably, in the data of Hand’s article,the configurations in the numerical solutions to Laplace’s equation differ: the left configuration is aligned, while the right is arranged in a brick-like pattern. The 2016 Jofnston’s article set different value of α, and the variable α, representing the ratio of intracellular to extracellular conductivities (g_i⁢l_/g_el_), is set at 1.6 on the left and 0.6 on the right, illustrating the impact of this parameter on the simulation outcomes. E, experiment; PE, partial experiment; N, numerical simulation; g_it_, intracellular transverse conductivities; g_el_, extracellular longitudinal conductivities; g_i⁢l_, intracellular longitudinal conductivities.

**Fig. 4.**
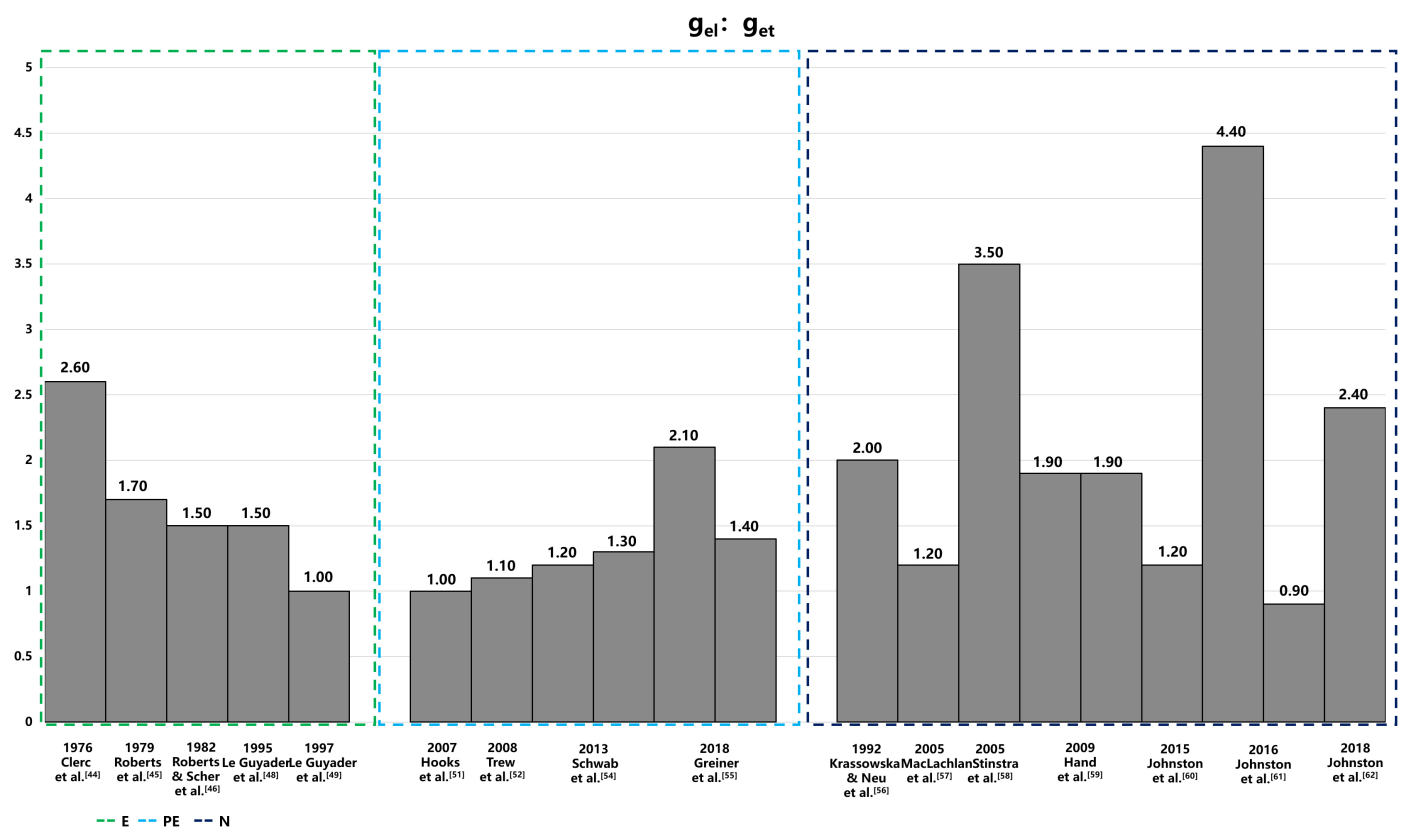
**Anisotropic ratios of extracellular conductivity from experiments and numerical simulations**. This figure displays the anisotropic ratios of extracellular conductivity as determined through experimental measurements and numerical simulations. Notably, in the data of Hand’s article,the configurations in the numerical solutions to Laplace’s equation differ: the left configuration is aligned, while the right is arranged in a brick-like pattern. The 2016 Jofnston’s article set different value of α, and the variable α, representing the ratio of intracellular to extracellular conductivities (g_i⁢l_/g_el_), is set at 1.6 on the left and 0.6 on the right, illustrating the impact of this parameter on the simulation outcomes. E, experiment; PE, partial experiment; N, numerical simulation; g_et_, extracellular transverse conductivities; g_el_, extracellular longitudinal conductivities; g_i⁢l_, intracellular longitudinal conductivities.

The electrical conductivity of the intracellular and extracellular space in the heart has been extensively studied [42, 43, 44, 45, 46, 47, 48, 49]. Figs. 3,4 provide a summary of intracellular and extracellular conductivity values measured in experiments or derived through numerical simulations. Table 4 (Ref. [43, 44, 45, 46, 47, 48, 49, 50, 51, 52, 53, 54, 55, 56, 57, 58, 59, 60, 61, 62]) provides an overview of published conductivity values. While Johnston and Johnston [42] provide a comprehensive review of cardiac bidomain conductivity values obtained from both experimental and numerical studies, this section focuses exclusively on values from studies published more recently. In 2022, Greiner *et al*. [50] estimated the intracellular conductivities in normal and infarcted hearts using confocal microscopy and numerical simulations. In the control group, the longitudinal, transverse and normal conductivities were 4.19 mS/cm, 0.18 mS/cm and 0.06 mS/cm, respectively [50]. Correspondingly, in the MI group, values were 2.64 mS/cm, 0.24 mS/cm and 0.02 mS/cm, respectively [50].

**Table 4.** **Conductivity values from experimental measurements or calculated from numerical simulation (unit: mS/cm)**.

Year	Study	Method	Tissue type	g_i⁢l_	g_it_	g_in_	g_il_: g_it_	g_el_	g_et_	g_en_	g_el_: g_et_
1970	Weidmann *et al*. [43]	E	Sheep/calf RV	1.600	-	-	-	5.300	-	-	-
1976	Clerc [44]	E	Calf RV	1.700	0.190	-	8.900	6.300	2.400	-	2.600
1979	Roberts *et al*. [45]	E	Canine LV	2.800	0.260	-	10.800	2.200	1.300	-	1.700
1982	Roberts & Scher [46]	E	Canine LV	3.400	0.600	-	5.700	1.200	0.800	-	1.500
1987	Kleber & Riegger [47]	E	Rabbit RV	4.500	-	-	-	4.000	-	-	-
1995	Le Guyader *et al*. [48]	E	Canine A	2.000	0.230	-	8.400	2.900	1.900	-	1.500
1997	Le Guyader *et al*. [49]	E	Canine A	0.600	0.390	-	1.500	1.300	1.300	-	1.000
2007	Hooks [51]	PE	Rat LV	2.600	0.260	0.080	10.000	2.600	2.500	1.090	1.000
2008	Trew *et al*. [52]	PE	Porcine LV	3.500	0.400	0.100	8.800	3.500	3.100	1.400	1.100
2013	Bauer *et al*. [53]	PE	Rabbit LV	0.650	0.042	0.033	15.500	-	-	-	-
2013	Schwab *et al*. [54]	PE	Rabbit LV	-	-	-	-	2.600	2.200	1.300	1.200
			Rabbit LV MI	-	-	-	-	2.600	2.000	1.700	1.300
2018	Greiner *et al*. [55]	PE	Rabbit LV	-	-	-	-	3.600	1.700	1.000	2.100
			Rabbit LV MI	-	-	-	-	6.900	5.100	2.000	1.400
2022	Greiner *et al*. [50]	PE	Rabbit LV	4.200	0.180	0.060	23.300	-	-	-	-
			Rabbit LV MI	2.600	0.240	0.020	10.830	-	-	-	-
1992	Krassowska & Neu [56]	N	Canine	0.700	0.003	-	233.000	3.000	1.500	-	2.000
2005	MacLachlan *et al*. [57]	N	Canine	3.000	1.000	-	3.000	2.000	1.700	-	1.200
2005	Stinstra *et al*. [58]	N	-	1.600	0.005	-	32.000	2.100	0.600	-	3.500
2009	Hand *et al*. [59]	N	Murine V	1.000	0.030	-	33.000	3.000	1.600	-	1.900
			Murine V	1.400	0.030	-	47.000	3.000	1.600	-	1.900
2016	Johnston [60]	N	Porcine LV	2.400	0.350	0.080	6.900	2.400	2.000	1.100	1.200
2016	Johnston *et al*. [61]	N	Porcine LV	1.900	0.350	0.080	5.400	3.200	2.200	1.200	4.400
			Porcine LV	3.100	0.350	0.080	8.900	2.000	2.200	1.200	0.900
2018	Johnston *et al*. [62]	N	Porcine LV	2.400	0.240	0.100	10.000	2.400	1.600	1.000	2.400

E, experiment; PE, partial experiment; N, numerical simulation; A, atria; RV, right ventricle; LV, left ventricle; MI, myocardial infarction; g_i⁢l_, intracellular longitudinal conductivities; g_it_, intracellular transverse conductivities; g_in_, intracellular normal conductivities; g_el_, extracellular longitudinal conductivities; g_et_, extracellular transverse conductivities; g_en_, extracellular normal conductivities.

The measurement methods for conductivity are categorized into three distinct groups: values directly measured in experiments (E), values estimated from experimental data (PE), and values deduced from the theoretical models (numerical simulation [N]). The conductivity values obtained from these methods exhibit significant variability, both absolute value and anisotropic ratio, with intracellular conductivity showing particularly notable differences. The variations could be attributed to differences in experimental conditions, measurement techniques, and the biological diversity of species or cells sampled from various parts of the atrium or ventricle. Table 4, organizes conductivity values according to these categories. The values derived from theoretical models, in particular, display substantial variability due to different mathematical models and theoretical assumptions, which is especially evident in the anisotropic ratios of conductivities.

## 8. Factors Influencing CV and Conductivity Selection for Simulation Studies

There were significant variations in the longitudinal CV of the human heart between various clinical groups. For example, the CVs reported by Nishimura *et al*. [15] and Martin *et al*. [16] showed minimal variation, with means of 21.68 cm/s and 23.06 cm/s, respectively. Similarly, the CVs reported by Martin *et al*. [17] and Tung *et al*. [18] were closely matched at 29.0 cm/s and 30.77 cm/s, respectively. Despite some discrepancies among different research groups, there was considerable overlap in the observed ranges of CVs (see Fig. 1). The variation in CV values may be attributed to differences in the infarct tissue characteristics, including the gray zone and scar tissue among patients in different studies. The proportion of scar to LV mass and gray zone to LV mass have been reported to range from 9% to 20% and 4% to 19% respectively [63]. Another possible explanation for the differences is that the CV decrease is directly proportional to the fibrosis density, as observed on late gadolinium enhancement cardiovascular magnetic resonance imaging (LGE-CMR) in patients with ischemic cardiomyopathy (ICM) [64, 65, 66]. Thus, CV measurements in the isthmus and outer loop may vary among patients.

Frontera *et al*. [14] reported a mean longitudinal CV of 44 cm/s in the isthmus, which is faster than the values ranging from 21.68 to 30.77 cm/s reported by other groups [15, 16, 17, 18]. This discrepancy could be attributed to measurement conditions, as the CV in [14] was assessed during sinus rhythm. Studies have demonstrated an inverse relationship between CV and pacing cycle length (PCL) [67, 68]. For example, a CV of 55.00 cm/s was recorded at a PCL of 600 ms, compared to a significantly slower CV of 32.00 cm/s at a PCL of 250 ms [67]. In clinic settings, the PCL during sinus rhythm typically exceeds 600 ms, contrasting with the median CL of VT, which is about 300 ms [15, 69]. Thus, the CV in the isthmus during VT is expected to be slower than the CV measured during sinus rhythm, which may explain the faster longitudinal CV in the isthmus reported by Frontera *et al*. [14] compared to those documented by other studies [15, 16, 17, 18].

A notable observation reported by Frontera *et al*. [19] is that the mean longitudinal CV in the isthmus during VT was significantly faster than the values reported in other studies presented in Table 1, including those for the outer loop. Notably, the CV in the isthmus was comparable to those reported in experimental studies listed in Table 2. It should be noted that the CV in slow condition corridors, which are part of the isthmus, was measured at 8.30 cm/s during sinus rhythms, indicating the presence of slow conduction regions in the isthmus. However, the reasons for the unusually fast CV in the isthmus during VT remain unclear, and the original paper did not provide any explanation for this observation [19].

The unusually high mean longitudinal CV in the isthmus during VT, as indicated in Frontera *et al*. [19], may be attributed to several factors. These include the choice of signal type, whether bipolar or unipolar, as well as the specific techniques used for measurement, such as the local activation time (LAT) difference between electrode pairs or the peak amplitude of the bipolar electrogram (EGM). Moreover, the criteria set for signal annotation play a crucial role, where inaccuracies in annotation could notably skew CV estimations. Therefore, it is essential to systematically analyze the signal annotation process, observing how near-field and far-field signals are distinguished in both bipolar and unipolar modalities. Such a review would aim to uncover any inconsistencies in annotation that could contribute to the observed discrepancies in CV values. By understanding these variances, work towards standardizing CV measurement techniques to enhance the accuracy and reliability of these critical readings in both clinical and experimental electrophysiology.

The mean longitudinal CV in the isthmus during VT, as measured in the study conducted by Hawson *et al*. [20], was slower compared to the CVs listed in Table 1. This discrepancy could stem from differences in measurement methodologies. Specifically, Hawson *et al*. [20] utilized the triangulation method, whereas most other studies predominantly employed the isopotential lines method. Previous research has demonstrated that CV measurements using the triangulation method in atria [70, 71] are generally slower than those obtained using the isopotential lines method [72]. Another possible explanation could be the varying definitions of the isthmus. In Hawson’s study [20], the isthmus was defined specifically as regions where local potentials occurred at 50%–70% of the annotation window within the isthmus, a narrower definition compared to other studies that considered the entire isthmus region [14, 16, 17, 19]. This difference in operational definitions could further account for the observed variations in CV measurements.

The slowing of CV in VT extends beyond simple conduction delay, it involves a complex interplay of factors such as the zig-zag phenomenon, as noted by Ciaccio *et al*. [24]. In the context of MI, the healing process involves fibrogenesis, which not only alters the myocardial architecture but also creates geometric constraints for the surviving, electrically active cells at the infarct border zones [73]. This process contributes to the thinning of the heart wall [74] and leads to the development of fibrotic areas with differing electrical conductivity compared to intact myocardial cell bundles. These changes result in the formation of narrow conducting channels, forcing the activation wavefront to navigate a zigzag path through these channels, effectively slowing the CV [32, 75]. Within these scarred regions following MI, surviving myocyte bundles often exhibit impaired conduction, characterized by slow and non-uniform electrical activity, primarily due to inadequate intercellular coupling [76]. These compromised conduction areas provide a fertile substrate for the emergence of reentrant circuits, a critical mechanism underlying VT [3, 6]. Thus, the zig-zag phenomenon represents a significant alteration in the path of electrical propagation due to structural changes post-MI, distinguishing it from mere slow conduction which might arise from altered electrophysiological properties without such pronounced anatomical modifications. This distinction is crucial for understanding the mechanisms that contribute to the complex electrophysiological landscape in VT following MI and underscores the need for targeted therapies that address these specific structural and functional changes.

The reductions in both longitudinal and transverse CVs during ischemia, are driven by multiple pathophysiological factors. Firstly, ischemia reduces ATP production due to limited oxygen supply [77], causing a shift to anaerobic respiration, which in turn impairs ion pump function in cardiac cells [78, 79]. This impairment alters ion gradients, particularly increasing intracellular Na^+^ and Ca^2+^ levels [78, 79], which disrupts action potential propagation and inhibits myocardial contractile function.

Furthermore, ischemia-induced acidosis, resulting from lactate accumulation, exacerbates the impairment of ion channel function, adversely impacting electrical conduction. These alterations contribute to heterogeneous changes in action potential duration (APD) [80]. Beyond the direct effects on ion channels, ischemia also triggers the downregulation and redistribution of gap junction proteins in both human and animal models [76], which diminishes coupling between muscle layers and exposes intrinsic electrophysiological heterogeneities [81]. Such changes in gap junction expression and function may independently enhance transmural APD heterogeneities [80]. Structural changes in the myocardium, such as fibrosis, physically disrupt the conduction pathways, further slowing CVs [32, 75]. Additionally, coupling between myocytes and fibroblasts in infarct hearts may further slow conduction [82, 83]. Collectively, these modifications result in a heterogeneous alteration of APD, reflecting the complex interplay between metabolic, structural, and electrophysiological factors under ischemic conditions.

Overall, the longitudinal CV at the entrance and exit of the isthmus demonstrated greater consistency when compared to similar measurements across the isthmus (Table 1), with a notable exception being Tung’s study [18], which reported a faster CV. This inconsistency may stem from differing definitions of the entrance and exit regions. In the aforementioned Tung *et al*. [18], the isthmus entrance and exit regions were defined based on the classic isthmus definition relative to tachycardia cycle length (TCL), categorized by timing alone. However, most other studies utilized the appearance of the common pathway delineated by activation mapping [14, 15, 16, 17, 19]. Interestingly, the CV in the outer loop measured during VT showed remarkable similarity in certain studies [15, 19], yet was significantly slower in Hawson’s study [20]. This discrepancy may also be attributed to differing measurement methods, as discussed above.

The longitudinal and transverse CVs of the human heart, as reported above (Table 2), show significant variation. Lang *et al*. [30] noted faster baseline CVs when compared to the observations published by Doshi *et al*. [29] and Anderson *et al*. [34], but with smaller longitudinal and transverse anisotropic ratios. Modeling cardiomyocyte activity through cylindrical equations, it was demonstrated that the CV is proportional to the square root of the cylinder diameter [84, 85]. Based on this model and the commonly observed anisotropy values of 5 to 10 in the intracellular space, the longitudinal and transverse anisotropic ratio reported by Lang *et al*. [30] may not align with the typical anisotropy range. Additionally, the findings of Doshi *et al*. [29] included only one patient, which limits the generalizability of the results. The values reported by Anderson *et al*. [34], approximating a recently measured longitudinal CV of 81 cm/s in areas with no visible fibrosis in patients with ischemic cardiomyopathy, likely provide a more accurate estimate for the normal human heart.

The anisotropic ratio of longitudinal and transverse CVs measured by de Bakker *et al*. [31, 32] in papillary muscle was significantly higher than the values reported in Table 2 for ventricular tissue. This discrepancy may be attributed to the well-ordered fiber orientation in papillary muscles [27]. Diffusion tensor imaging studies have also demonstrated that inclination angles in the papillary muscle closely align to angles of either –90° or 90°, indicating an apex-to-base orientation [27]. This more uniform fiber architecture in papillary muscles, as opposed to the more varied arrangement in ventricular tissue, may contribute to the higher anisotropic ratio of longitudinal and transverse CVs.

Moreover, prior studies suggest that the Purkinje network can deliver swift and synchronized electrical activation to the ventricular myocardium, which is critical for effective cardiac function [86, 87]. However, the uneven distribution of Purkinje fibers within the papillary muscles—characterized by a high concentration at the base and an absence at the apex—creates alternative conduction pathways for the rapid transmission of electrical signals along the length of the papillary muscles [88]. This unique arrangement of myocardial and Purkinje fibers within the papillary muscles leads to non-uniform conduction properties. Particularly, the transition from fast-conducting Purkinje fibers to slower-conducting myocardial fibers may create zones of delayed conduction, thus impacting the overall conduction velocities and exacerbating the anisotropic nature of the heart muscle, where electrical conduction varies directionally [89]. Although these structural and functional disparities are thought to increase conduction velocities and enhance anisotropic ratios longitudinally, they potentially predispose cardiac tissue to the formation of re-entrant circuits—a known substrate for ventricular fibrillation (VF) [90, 91, 92]. However, it is important to note that the relationship between these anatomical features and the electrophysiological properties of the diseased Purkinje system, seen in conditions such as in ischemic cardiomyopathy or Purkinje-related VF, remains poorly understood. The distinct organization of these fibers, especially in the presence of an uneven distribution of Purkinje fibers, could collectively be a significant factor in the genesis of Purkinje-related VF, which can be one explain on how such arrhythmias might initiate and be sustained within this specialized cardiac network.

Signal-averaged electrocardiography (SAECG), initially developed for noninvasive recording of His bundle potentials, now plays a pivotal role in detecting low-amplitude electrical activity in the myocardium. This technique detects slow conduction in ventricular areas, as indicated by late potentials. Clinical data from patients with healed myocardial infarction have underscored the utility of SAECG in risk stratification [93, 94]. The integration of SAECG into algorithms that merge both invasive and noninvasive indices significantly improves its effectiveness in identifying candidates for device therapy aimed primarily at preventing sudden cardiac death.

In a diseased state, the longitudinal CV generally decreases, as shown in most studies listed in Table 2, but the extent of this reduction varies considerably. Factors influencing these variations include the disease type, mapping method, and CV measurement method. A comprehensive review of the determinants of CV in clinical or experimental settings was published by Han *et al*. [84]. Furthermore, the anisotropic ratio of longitudinal and transverse CV undergoes significant changes during early ischemia and in nonischemic end-stage heart failure states compared to the control state, whereas only minor changes were observed in the DCM state (Tables 2,3).

Several histological studies have reported a reduction connexin 43 (Cx43) expression—of over 40%—in the sub-epicardial and sub-endocardial myocardium of the left ventricle in heart failure patients, along with a disrupted distribution of Cx43 [95, 96]. In addition, the presence of interstitial fibrosis has been linked to discontinuous propagation and spatial dispersion of CV in heart failure patients [37]. Similar downregulation and lateralization of Cx43, along with a reduction in peak sodium current compared to normal values, have also been reported in patients with ischemic heart disease [95, 96, 97]. In cases of cardiac hypertrophy, an increase in cell size, elevated levels of Cx43, and lateralization of Cx43 contribute to changes in CV and the anisotropic ratio [95, 96, 97].

The conductivities listed in Table 4 exhibit significant variability depending on the methods used for measurement or calculation. Conductivities measured through experiment or partial experiment tend to be more consistent than those derived from numerical simulations, with the exception of the extremely small value reported by Le Guyader *et al*. [49] and the exceptionally large value reported by Greiner *et al*. [50]. Drawing on the finding of Eisenberg [85] and the most commonly measured CVs in experiments, an anisotropy range of 5 to 16 could be a suitable choice for cardiac modeling under both normal or diseased conditions. Furthermore, it is essential to consider the underlying mechanisms of reentry formation. Specifically, the product of the electrical signal propagation velocity and the refractory period must be smaller than the length of the reentrant pathway. Setting excessively high CV in normal or infarcted tissue may lead to a reduced inducibility rate or render models representing clinical VT scenarios unable to initiate reentry. Conversely, excessively slow CV settings may induce reentry in locations different from the actual VT site. These considerations underscore the importance of aligning numerical parameters with physiological principles when modeling cardiac electrophysiology.

Large variations in CV have been observed in both experimental and clinical settings. Consequently, the CV values used in personalized cardiac modeling also show considerable variation [11, 98, 99, 100, 101, 102]. This variability persists even under the same disease conditions, such as in simulations related to infarct-related VT [100, 103] and atrial fibrillation [103, 104]. It is challenging to determine the optimal CV value because it must be tailored to individual patient characteristics and specific diseases [103, 104]. Several studies have investigated how different CV settings affect the accuracy of simulations for clinical VT and atrial fibrillation (AF) [103, 104]. The findings indicate that variations in CV significantly affect the reentry characteristics, particularly in AF [105]. However, the impact of CV adjustments in computational models of VT in post-infarction patients appears to be relatively minor [103]. Future studies should investigate how varying CV settings in computational models of other heart diseases may influence simulation outcomes when validated against clinical measurements.

## 9. The Role of Repolarization Dispersion in the Pathophysiology of VT

The role of repolarization dispersion in the pathophysiology of VT is an area of growing interest and significance [5, 106]. Repolarization dispersion refers to the variation in the duration of the repolarization phase across different regions of the ventricular myocardium. This heterogeneity arises from several factors, including differences in action potential duration, the presence of myocardial scar tissue, and alterations in ion channel function, it also can be exacerbated by sympathetic stimulation, either physiologically or pharmacologically [107, 108].

The importance of repolarization dispersion stems from its ability to create a substrate conducive to reentrant arrhythmias [109]. In VT, areas with prolonged repolarization can lead to early afterdepolarizations (EADs) [110, 111] and delayed afterdepolarizations (DADs) [111, 112, 113], which are known triggers for the initiation of malignant ventricular arrhythmias. Additionally, regions of the myocardium that repolarize prematurely can form the unidirectional block necessary for the development of reentry circuits, a hallmark of sustained VT [114, 115, 116]. Moreover, repolarization dispersion is amplified under pathological conditions such as ischemia, heart failure, and structural heart disease, further increasing susceptibility to VT [77]. Current research, including experimental models and clinical studies, suggests that interventions aimed at reducing repolarization dispersion, such as ion channel modulators [78, 79] or targeted ablation, may provide therapeutic benefit in managing and preventing VT. By focusing on the mechanisms underlying repolarization dispersion and its relationship with VT, future investigations can unveil novel insights into arrhythmogenesis. This could lead to enhanced risk stratification and the development of more effective treatment strategies for patients with ventricular arrhythmias.

## 10. Advanced Techniques for Measuring and Calculating CV

Accurately measuring cardiac CV is crucial for simulating the heart’s electrical activity. Two basic properties are used to measure CV: (1) the distance traveled by an electrical pulse within a specific time period, and (2) the time taken for an electrical pulse to travel a certain distance. Ideally, to account for the heart’s anisotropic properties, multiple recording sites are necessary to accurately reflect the longitudinal and transverse CV [84]. Early experiments often used plunge-needle electrodes to obtain high-resolution activation maps, myocardial transmural data, and multi-point measurements [117, 118]. In laboratory settings, optical mapping at high spatiotemporal resolution is commonly used to record the electrical activity of isolated mammalian hearts [2, 35]. In clinical environments, catheter-based mapping is the most prevalent method for measuring CV. This technique utilizes catheters equipped with at least two electrodes to collect intracardiac EGMs from multiple locations within the target chamber [84].

The integration of microelectrode technology into catheter design represents a transformative advancement in electrophysiological mapping and ablation strategies throughout the heart, notably enhancing ventricular substrate characterization [119]. Equipping catheters with mini-electrodes (ME) at the ablation tip has proven to significantly enrich data collection, which is critical for detecting and analyzing cardiac signals [119]. These microelectrodes provide superior spatial resolution, facilitate the precise identification of conducting bundles not only within the cavo-tricuspid isthmus (CTI) but also in more complex ventricular substrates [120]. Furthermore, the mini electrodes positioned at the catheter’s ablation tip offer more accurate identification of the electrical properties of the tissue directly beneath the ablation surface, enabling a more detailed and precise mapping of cardiac electrical activity [121]. The ME technology has also shown significant efficacy in identifying intramural lesions that are often obscured by layers of viable myocardium, thereby enhancing the identification of complex arrhythmogenic substrates such as intramural reentry, which are critical for effective ablation [18, 119].

Recent studies have highlighted the benefits of ME technology in providing detailed insights into ventricular electrophysiological properties. For instance, the use of ME catheters has been shown to improve the detection of pathway potentials and localize ablation sites with greater accuracy, especially in the context of pulmonary vein isolation (PVI). Acute success rates and procedural outcomes, such as the extent of low-voltage areas (LVA) [121], were comparable to those achieved with standard contact force-sensing catheters, underscoring the ME technology’s efficacy and reliability.

Moreover, during ablation procedures, ME catheters have demonstrated a significant reduction in signal amplitude, which serves as a reliable indicator of tissue viability post-ablation [122]. This capability is critical for distinguishing between viable and non-viable tissues [123], thereby optimizing the effectiveness of radiofrequency applications and minimizing unnecessary ablations [122]. Such capabilities enable more precise voltage-directed ablation strategies, which have been validated by both animal models and clinical reports [124, 125]. Notably, these strategies are crucial for managing arrhythmias, as they help in accurately highlighting gaps within linear ablation lines and in identifying viable ablation targets.

Standard cardiac electroanatomic mapping systems are capable of recording both unipolar and bipolar EGMs. However, due to the heart’s complex electrical activity, a single electrode cannot provide accurate information about both the velocity and direction of the wavefront. Therefore, multiple measurements taken from different locations are generally required to build an activation map, allowing for an improved understanding of the spatiotemporal characteristics of the heart’s electrical activity [84]. For example, one type of catheter commonly used in clinical settings is the HD multi-electrode Grid catheter (Abbott, Abbott Park, IL) [84]. This configuration features 16 electrodes arranged in a 4 × 4 grid pattern, which can capture high-density electrical signals across a large surface area of the heart. This allows for more accurate activation mapping and localization of arrhythmias. Beyond the HD Grid, other catheters also offer high-resolution mapping capabilities. For instance, the Orion catheter (Boston Scientific, Cambridge, MA) features an array of 64 mini-electrodes distributed across 8 splines, enhancing detailed mapping through both magnetic and impedance detection [126]. Similarly, Biosense-Webster has developed advanced tools such as the Optrell, which includes 48 mini-electrodes over 6 splines, and the OctaRay, equipped with 48 electrodes on 8 radial splines [127]. These systems not only provide comprehensive coverage but also facilitate precise localization of electrical activity, contributing significantly to the effective treatment of arrhythmias [128].

In the context of measuring CV, both bipolar and unipolar electrode configurations offer distinct advantages and challenges. Bipolar configurations, which utilize two closely spaced electrodes, excel in reducing electrical interference and enhancing signal clarity due to their ability to create a localized current dipole [129]. This setup enhances the signal-to-noise ratio (SNR), making it particularly effective for detecting specific, localized changes in CV within a small region of the heart [129]. This precision is crucial for accurately mapping arrhythmogenic areas and assessing the efficacy of therapeutic interventions, as it enables the detection of specific, localized changes in CV within a small region of the heart [129].

Conversely, unipolar configurations employ a single electrode to measure potential relative to a distant reference, providing a broader view of the heart’s electrical activity [84, 129]. While this setup captures data from a larger area, it is more susceptible to noise and may offer less precise information about the velocity and direction of electrical wavefronts, potentially leading to less accurate CV measurements [84, 126, 127, 128, 129]. However, its ability to monitor extensive myocardial activity can be invaluable for initial diagnostic assessments and for understanding overall heart function. Thus, while bipolar configurations are preferred for detailed, localized studies, unipolar setups are more suitable for broader, general assessments, making the choice between them dependent on the specific clinical or research needs.

In clinical settings the identification of CV typically involves traditional methods. Regions of slowed conduction are identified through the detection of abnormal EGMs and/or isochronal crowding on LAT maps [20, 130, 131]. While this method provides an indirect assessment of CV, the density of isochrones on the visual map greatly depends on the selected time step interval for the isochronal map, and variations in the time step interval may impact the final CV value. Using isochronal maps to measure the distance traveled at fixed time intervals effectively estimates wavefront curvature and aids in noise discrimination [132, 133]. However, this technique requires high-resolution data and absolute membrane potential measurements, restricting its applicability to optical mapping in clinical settings. Alternatively, the ACVM algorithm utilizes a triangulation method that enables CV estimation from an arbitrary set of points on the surface without imposing significant constraints on their spacing or distribution [20, 133]. While it directly employs LAT and produces superior clinical and experimental data, it is sensitive to errors in activation time differences greater than 3 ms [20, 133].

The occurrence of near-field and far-field false annotations within the isthmus, entrance, and exit regions of VT circuits poses significant challenges. Current mapping techniques may lack the resolution necessary to fully characterize these regions [32, 134, 135]. One major limitation is the inadequate resolution of existing mapping technologies, which fails to capture the intricate activation patterns in thin layers of surviving endocardial fibers over densely infarcted zones [134]. This deficiency hinders the accurate differentiation between near-field and far-field signals, leading to potential misannotation. The complex architecture of VT circuits, as recent high-density mapping studies have revealed, presents additional challenges in accurately annotating signals within the isthmus, entrance, and exit regions [23]. The presence of multiple entrance and exit sites, dead ends of activation, and regions of activation within dense scar tissue further complicates the identification of near-field and far-field signals [23, 134]. Additionally, the dynamic nature of VT circuits, including the region of poorly-coupled fibers [136] that may conduct multiple VTs in different directions [17], further increases the risk of misannotation. These complexities underscore the need for advancements in mapping technology to enhance resolution and accuracy in characterizing VT circuits.

Exploring the effect of electrode configuration on activation detection variability, Takigawa *et al*. [137] analyzed the impact of electrode size and inter-electrode spacing on the precision of gap detection and the reduction of far-field signal contributions in AF EGMs. They reported an electrode configuration of 0.3 mm with 0.1 mm spacing between electrodes as nearly optimal [137]. This setup significantly improved the delineation of true local activation times within AF recordings and reduced intraobserver variability in identifying local activation times to zero [137].

## 11. Conclusions

The reduction of CV plays a pivotal role in the initiation and maintenance of reentrant arrhythmias. Therefore, it is essential to examine a range of CV values under both physiological and pathological conditions. The review synthesizes findings on CV values, anisotropic ratios, as well as longitudinal and transverse conductivity from clinical studies and animal models. Despite observing significant variations in these metrics across different studies, the data provide crucial insights that guide the selection of CV and conductivity values in cardiac modeling and other applications. It is important to ensure that the chosen CV value is appropriate for the specific disease context and be validated through cardiac modeling using clinical measurements, such as ECG [138], arrythmia inducibility [63], arrythmia mapping, or the identification of relevant ablation targets [11, 139].

## Abbreviations

CV, conduction velocity; CV_l_, longitudinal conduction velocity; CV_t_, transverse conduction velocity; CV_n_, normal conduction velocity; VTs, ventricular tachycardias; SD, standard deviation; ACVM, automated conduction velocity mapping; OL, outer loop; MI, myocardial infarction; DCM, dilated cardiomyopathy; E, measured entirely through experiments; PE, estimated from experimental data; N, deduced from theoretical models; LGE-CMR, late gadolinium enhancement cardiovascular magnetic resonance imaging; ICM, ischemic cardiomyopathy; LAT, local activation time; SCinOL, slow conduction in outer loop; VSCIB, very slow conduction in isthmus barriers; SR, sinus rhythm; CAD, coronary artery disease; NIC, end-stage; HF, heart failure; ECM, electrograms; PCL, pacing cycle length; APD, action potential duration; VF, ventricular fibrillation; SAECG, signal-averaged electrocardiography; EADs, early afterdepolarizations; DADs, delayed afterdepolarizations; CTI, cavo tricuspid isthmus; SNR, signal-to-noise ratio; ME, mini-electrodes; PVI, pulmonary vein isolation; LVA, low-voltage areas.
